# Artificial intelligence in obstetrics and gynecology: Evaluating ChatGPT and Google Gemini in answering patient questions

**DOI:** 10.1002/ijgo.70622

**Published:** 2025-10-28

**Authors:** Madeline West, Amir Alsaidi, Rohail Siddiqi, Fatima Sayyed, Rachael Counts, Lauren Quinto, Nicholas Stansbury

**Affiliations:** ^1^ Paul L. Foster School of Medicine Texas Tech University Health Sciences Center El Paso Texas USA; ^2^ Department of Obstetrics & Gynecology The University of Texas Health Science Center at San Antonio San Antonio Texas USA

**Keywords:** artificial intelligence, ChatGPT, Google Gemini, gynecology, obstetrics, patient education

## Abstract

**Introduction:**

To evaluate the accuracy and completeness of responses across common obstetrical and gynecologic topics generated by the large language models (LLMs) ChatGPT and Google Gemini, which have become increasingly popular for patients seeking medical information before physician consultations.

**Methods:**

Ten topics were identified, five obstetrical (prenatal labs, extended carrier screen, treatments for nausea and vomiting in pregnancy, gestational diabetes, and trial of labor after cesarean section) and five gynecologic (polycystic ovary syndrome, pelvic inflammatory disease, cervical smears, mammograms, and birth control). For each condition, ChatGPT generated five of the most frequently asked patient questions, which were then presented separately to ChatGPT and Google Gemini. Board‐certified Obstetrics and Gynecology physicians evaluated the responses using Likert scales for accuracy (1–6) and completeness (1–3).

**Results:**

Acceptable response criteria were defined as an accuracy score of 5 or greater (“nearly all correct”) and a completeness score of 2 or greater (“adequately complete”). Most responses from both models met these thresholds. Wilcoxon signed‐rank tests demonstrated statistically significant differences in accuracy and completeness between models (*P* < 0.05). Inter‐rater agreement was measured using intraclass correlation coefficients. For obstetrical topics, ChatGPT scored −0.047 (completeness) and 0.112 (accuracy), whereas Google Gemini scored 0.367 and 0.205, respectively. For gynecologic topics, ChatGPT scored 0.328 and 0.20, compared with Google Gemini at 0.151 and −0.08.

**Conclusion:**

Both LLMs provided largely accurate and complete responses to patient questions. ChatGPT demonstrated stronger outcomes overall, suggesting potential utility in patient education; however, patients should confirm online information with physicians given the limitations of LLMs.

## 1 INTRODUCTION

Access to accurate medical resources remains restricted by paywalls and technical jargon. Thirty‐five percent of adults in the USA have attempted to self‐diagnose using online resources, suggesting that many patients turn to the web as their choice for medical advice.^1^ Furthermore, most of patient‐education articles had a median readability score above the recommended level (sixth to eighth) set by national guidelines.^2^ This is relevant to the field of obstetrics and gynecology because of the complex aspects of pregnancy and childbirth, and diagnostic and treatment variabilities.^3^, ^4^

In recent years, artificial intelligence (AI) has witnessed rapid advancements and gained massive popularity among users, and medicine is not an exception. These AI tools, including Large Language Models (LLMs) such as ChatGPT and Google Gemini, have introduced innovative methods for patients to seek medical answers. Their popularity could be due to their easy access, user‐friendly platform, and their use of a question‐and‐answer format. In medicine, these chatbots offer reasonably accurate responses and are becoming a “first‐line” resource, particularly for patients who are hesitant to consult a provider.^3^, ^5^, ^6^

LLMs are not only limited to answering queries. Kung et al.^7^ examined ChatGPT's performance in the US Medical Licensing Examination (USMLE), where it scored above the passing threshold on all three STEPs, indicating strong potential for LLMs in the medical field. Allahqoli et al.^8^ further supported LLM performance in obstetrics and gynecology, as ChatGPT correctly managed 90% of clinical OB/GYN vignettes. Clinically, both Baeta et al.^9^ and Malakouti et al.^10^ reported promising potential for AI models' guidance in maternal hemorrhage scenarios; however, they emphasized that variations were also observed.

However, concerns have also arisen regarding the accuracy, reliability, transparency, and safety of these models.^5^, ^11^ Pavone et al.^5^ highlight that these AI‐generated responses are not peer‐reviewed, lack citations, and are based on training data rather than current clinical guidelines. Barrera et al.^12^ further support these concerns in their systematic review, demonstrating that while LLMs are capable of diagnosing polycystic ovary syndrome (PCOS), they used standardized diagnostic criteria of PCOS a third of the time. These AI lapses are worrying, particularly when used as the primary source for medical information, potentially leading to delayed care and uninformed health decisions.^6^

Given the widespread use of LLMs among patients, it is essential to evaluate the performance of different chatbots in addressing various OB/GYN topics. The purpose of this study is to assess the accuracy and completeness of the responses generated by ChatGPT and Google Gemini to patient questions regarding common OB/GYN topics.

## 2 MATERIALS AND METHODS

ChatGPT (version 3.5) was prompted to generate the five most common obstetrical topics, and five of the most common gynecologic topics searched on the web. In Obstetrics, the five topics were prenatal labs, extended carrier screening, treatments for nausea and vomiting during pregnancy, gestational diabetes, and trial of labor after cesarean section. On the other hand, in gynecology, the five topics were PCOS, pelvic inflammatory disease (PID), cervical smears, mammograms, and birth control. Each topic was then introduced to the free version of ChatGPT (v.3.5) and it was prompted to generate the five most frequently asked questions online about that topic.

The questions generated by ChatGPT were presented separately to both models. Two board‐certified OB/GYNs assessed each model's responses and evaluated them for completeness and accuracy using the Likert Scale. For completeness, the scale ranged from 1 to 3: (1) incomplete response, (2) adequate response, or (3) comprehensive and above‐expectation response. For accuracy, the scale ranged from 1 to 6, with each grade represented as follows: (1) completely incorrect, (2) more incorrect than correct, (3) equally correct and incorrect, (4) more correct than incorrect, (5) nearly all correct, or (6) completely correct. To be deemed acceptable, answers needed to achieve a score of at least 2 for “adequately complete” in completeness and 5 for “nearly all correct” in accuracy.

Then, the mean score for accuracy and completeness of each topic was calculated. Additionally, the mean scores for all questions across every topic were computed. Finally, the inter‐rater reliability for both metrics was assessed by calculating the intraclass correlation coefficient.

This study was non‐regulated research, so institutional review board approval was not required.

## 3 RESULTS

### 3.1 Completeness

**FIGURE 1:**
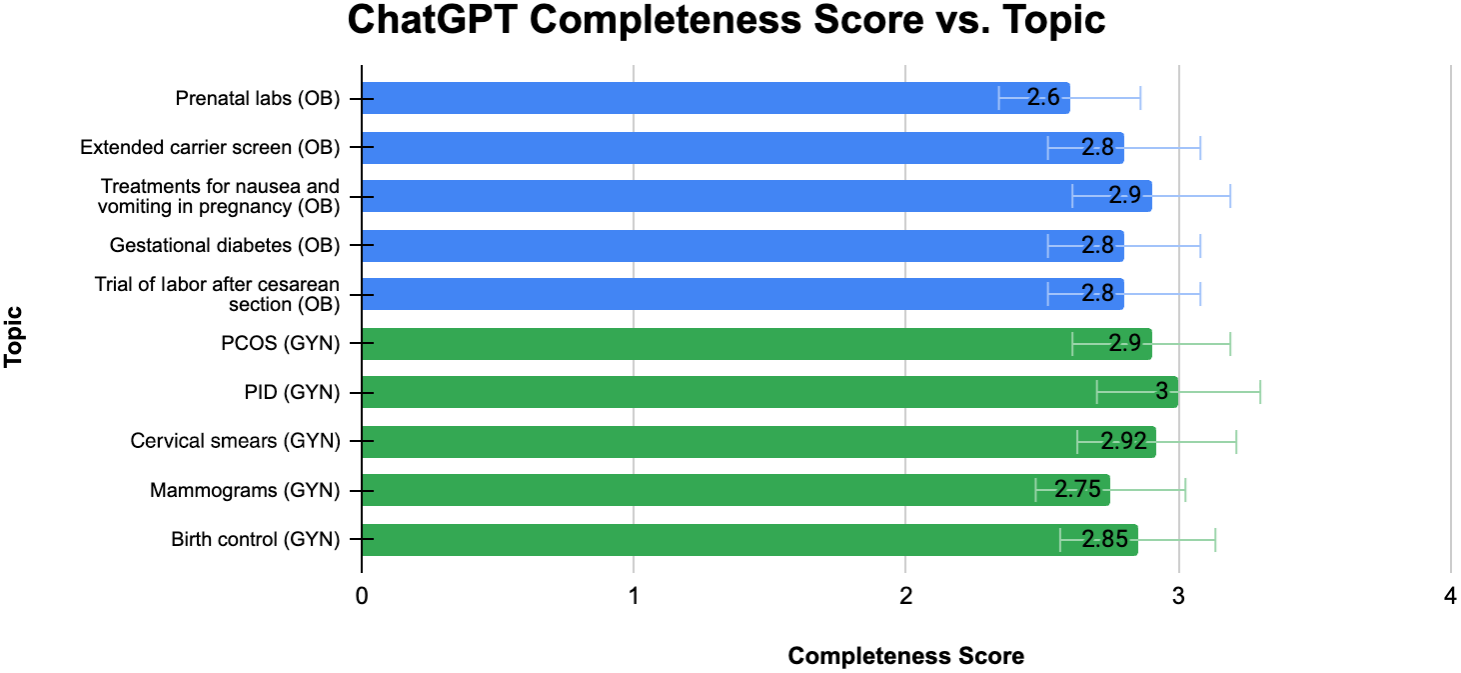
ChatGPT completeness average rating per topic.

**FIGURE 2:**
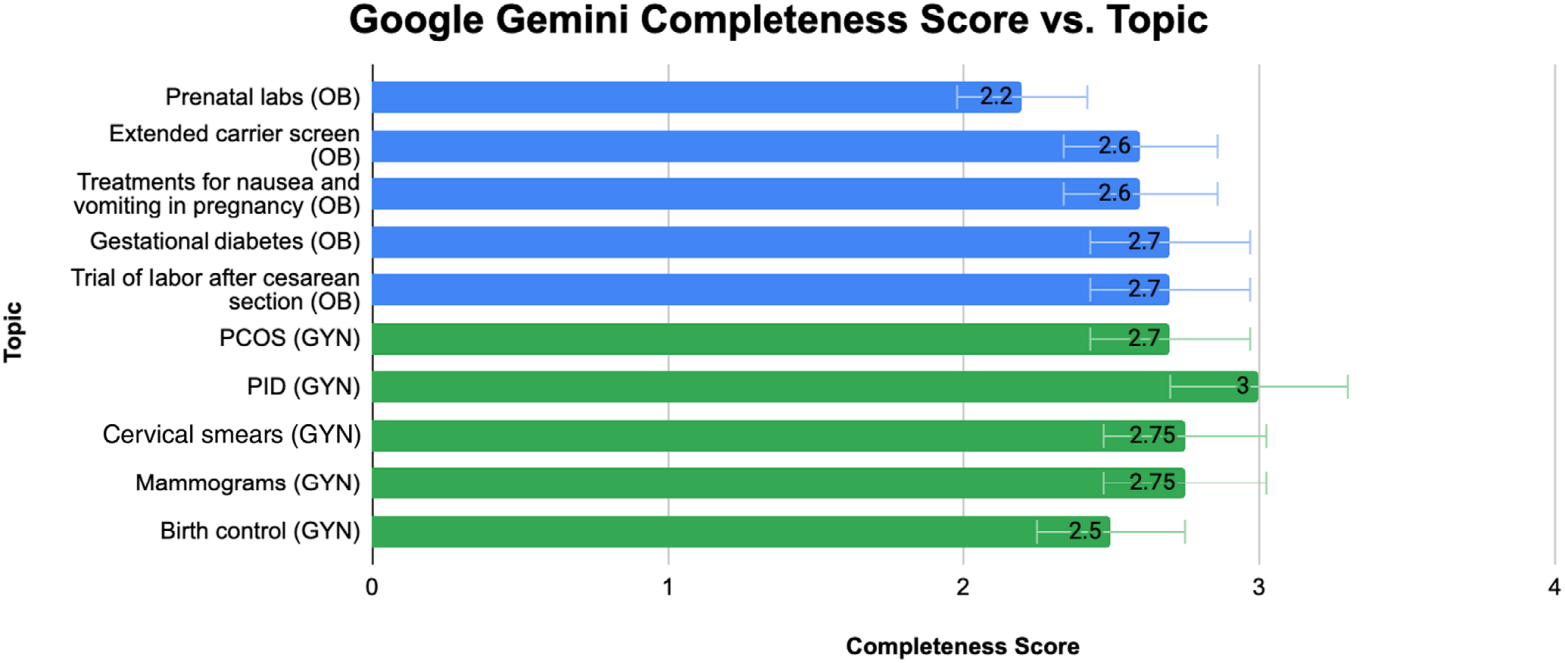
Google Gemini completeness average rating per topic.

Of the five selected topics in obstetrics, ChatGPT's average scores per topic were as follows: prenatal labs, 2.6; extended carrier screen, 2.8; treatments for nausea and vomiting in pregnancy, 2.9; gestational diabetes, 2.8; and trial of labor after cesarean section, 2.8. The overall average score for ChatGPT's completeness was 2.78. Conversely, Google Gemini's average scores per topic were: prenatal labs, 2.2; extended carrier screen, 2.6; treatments for nausea and vomiting in pregnancy, 2.6; gestational diabetes, 2.7; and trial of labor after cesarean section, 2.7. The overall average score for Google Gemini's completeness was 2.56. All responses from both models were considered thorough and met expectations. A Wilcoxon test was performed to compare the scores of each model, yielding a *P* value less than 0.05, indicating a statistically significant difference between ChatGPT and Google Gemini. Both models' ratings per topic are displayed in Figures 1 and 2, and in Table 1.

**TABLE 1:** Mean completeness scores of responses of each Large Language Model per question topic.

Diagnosis	OB completeness		Diagnosis	GYN completeness	
	ChatGPT	Google Gemini		ChatGPT	Google Gemini
Prenatal labs	2.6	2.2	PCOS	2.9	2.7
Extended carrier screen	2.8	2.6	PID	3	3
Treatments for nausea and vomiting in pregnancy	2.9	2.6	Cervical smears	2.92	2.75
Gestational diabetes	2.8	2.7	Mammograms	2.75	2.75
Trial of labor after cesarean section	2.8	2.7	Birth control	2.7	2.5
Overall	2.78	2.56	Overall	2.85	2.74
*P* value	<0.05		*P* value	<0.05	

Abbreviations: GYN, gynecology; OB, obstetrics; PID, pelvic inflammatory disease; PCOS, polycystic ovary syndrome.

Of the five selected topics in gynecology, ChatGPT's average scores per topic were as follows: PCOS, 2.9; PID, 3; cervical smears, 2.92; mammograms, 2.75; and birth control, 2.7. The overall average score for ChatGPT's completeness was 2.85 ± 0.36. Conversely, Google Gemini's average scores per topic were: PCOS, 2.7; PID, 3; cervical smears, 2.90; mammograms, 2.70; and birth control, 2.50. The overall average completeness score for Google Gemini was 2.74 ± 0.44. All responses from both models were deemed comprehensive and met expectations. A Wilcoxon signed‐rank test was conducted and resulted in a *P* value less than 0.05, indicating a statistically significant difference between ChatGPT and Google Gemini. Both models' ratings per topic are displayed in Figures 1 and 2, and in Table 1.

The average inter‐rater agreement score for completeness for each model was determined using a 95% confidence interval. ChatGPT earned agreement scores of −0.047 (no agreement) in obstetrics and 0.301 (fair agreement) in gynecology. In comparison, the agreement scores for Google Gemini were 0.367 (fair agreement) in obstetrics and 0.151 (slight agreement) in gynecology.

### 3.2 Accuracy

**FIGURE 3:**
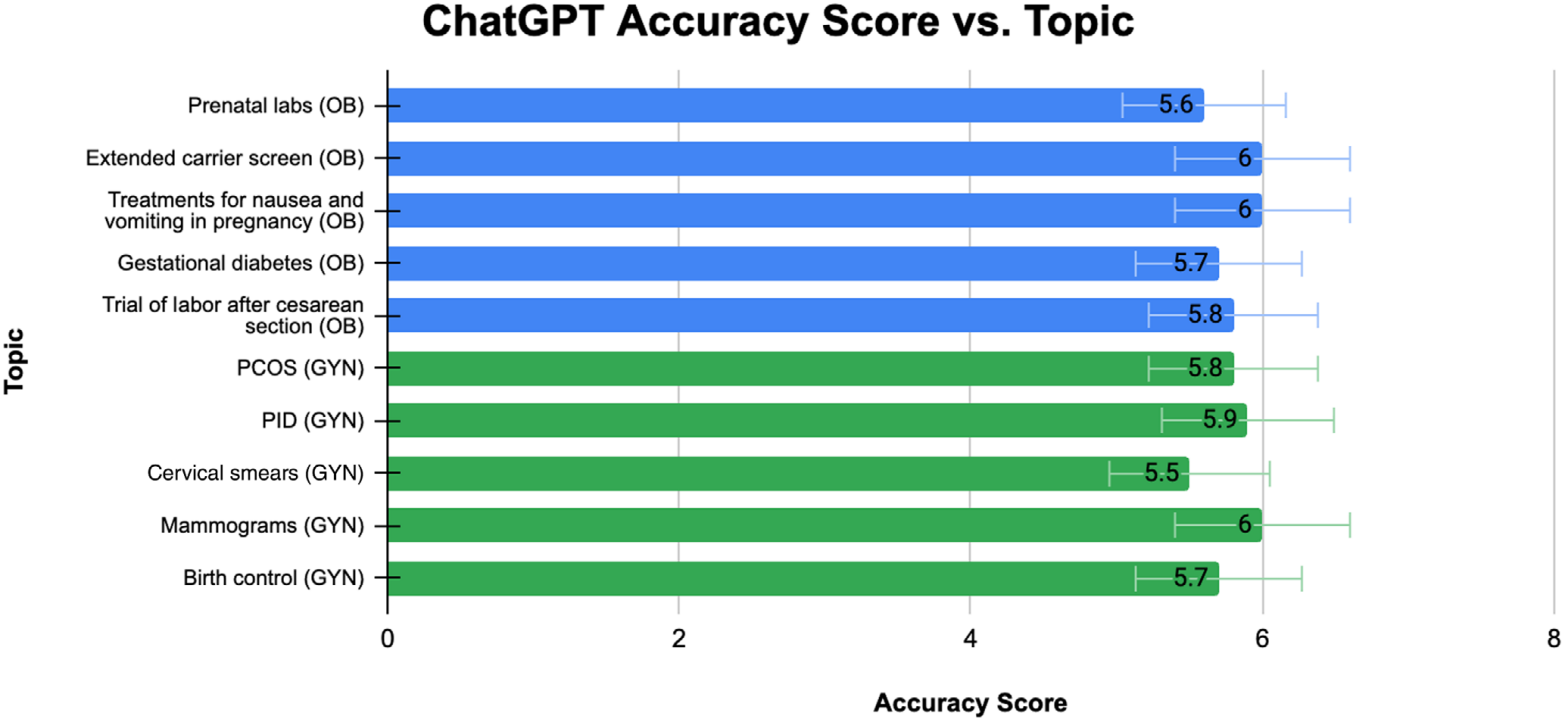
ChatGPT accuracy average rating per topic.

**FIGURE 4:**
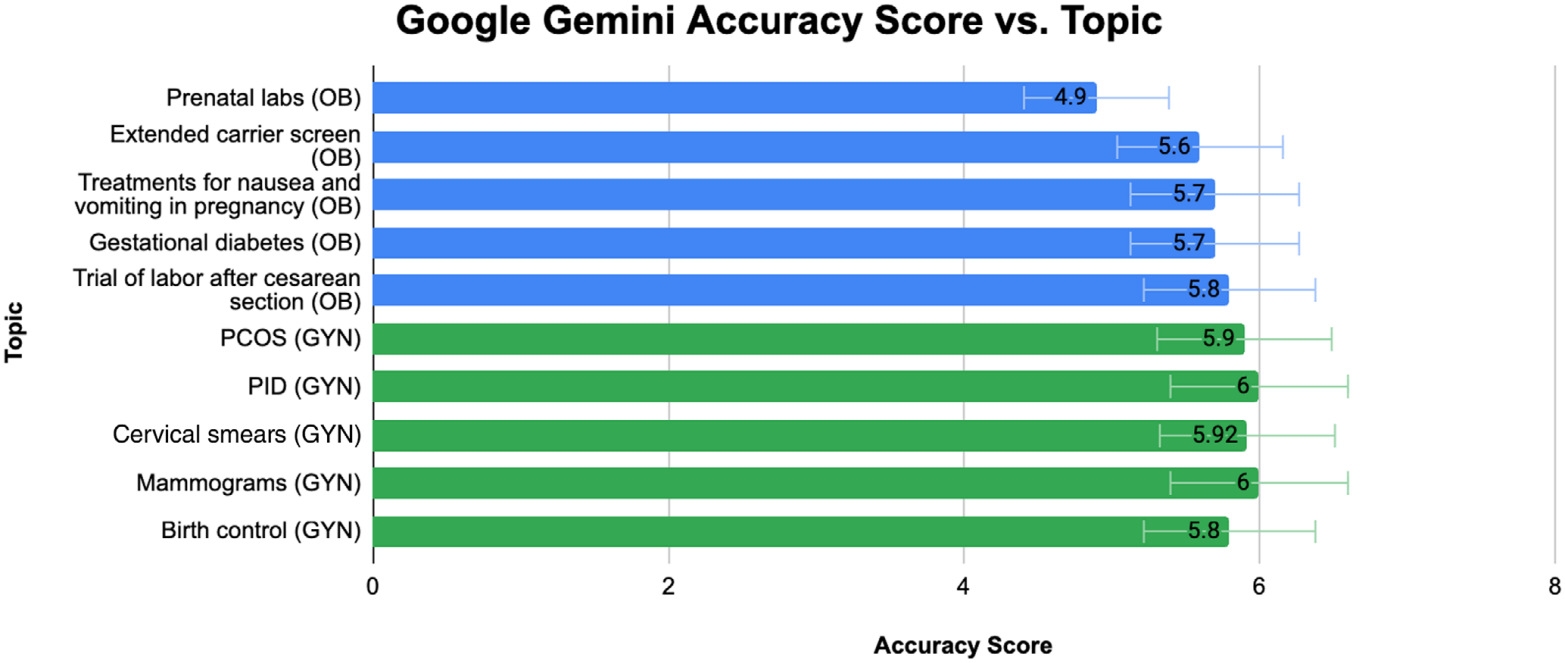
Google Gemini accuracy average rating per topic.

Of the five selected topics in obstetrics, ChatGPT's average scores per topic were as follows: prenatal labs, 5.6; extended carrier screen, 6.0; treatments for nausea and vomiting in pregnancy, 6.0; gestational diabetes, 5.7; and trial of labor after cesarean section, 5.8. The overall average score for ChatGPT's completeness was 5.82. In comparison, Google Gemini's average scores per topic were: prenatal labs, 4.9; extended carrier screen, 5.6; treatments for nausea and vomiting in pregnancy, 5.7; gestational diabetes, 5.7; and trial of labor after cesarean section, 5.8. The overall average score for Google Gemini's completeness was 5.54. Overall, both chatbots produced accurate responses, except for prenatal labs, where Google Gemini did not meet the acceptable answer criteria. A Wilcoxon signed‐rank test was performed and yielded a *P* value less than 0.05, which indicates a statistically significant difference between ChatGPT and Google Gemini. Both models' ratings per topic are displayed in Figures 3 and 4, and in Table 2.

**TABLE 2:** Mean accuracy scores of responses of each Large Language Model per question topic.

Diagnosis	OB accuracy		Diagnosis	GYN accuracy	
	ChatGPT	Google Gemini		ChatGPT	Google Gemini
Prenatal labs	5.6	4.9	PCOS	5.8	5.9
Extended carrier screen	6	5.6	PID	5.9	6
Treatments for nausea and vomiting in pregnancy	6	5.7	Cervical smears	5.5	5.92
Gestational diabetes	5.7	5.7	Mammograms	6	6
Trial of labor after cesarean section	5.8	5.8	Birth control	5.7	5.8
Overall	5.82	5.54	Overall	5.78	5.92
*P* value	<0.05		*P* value	<0.05	

Abbreviations: GYN, gynecology; OB, obstetrics; PID, pelvic inflammatory disease; PCOS, polycystic ovary syndrome.

Of the five selected topics in gynecology, ChatGPT's average scores per topic were: PCOS, 5.8; PID, 5.9; cervical smears, 5.5; mammograms, 6.0; and birth control, 5.70. The overall average score for ChatGPT's accuracy was 5.78 ± 0.17. In comparison, Google Gemini's accuracy scores were: PCOS, 5.90; PID, 6.0; cervical smears, 5.92; mammograms, 6.0; and birth control, 5.80. The overall average score for Google Gemini's accuracy was 5.92 ± 0.07. Overall, both ChatGPT and Google Gemini generated responses that were considered accurate, providing more correct than incorrect answers across all five topics. A Wilcoxon signed‐rank test was conducted and yielded a *P* value less than 0.05, indicating a statistically significant difference between ChatGPT and Google Gemini. Both models' ratings per topic are displayed in Figures 3 and 4, and in Table 2.

The average inter‐rater agreement score for accuracy for each model was determined using a 95% confidence interval. ChatGPT earned accuracy agreement scores of 0.112 (slight agreement) for obstetrics and 0.21 (fair agreement) for gynecology. In comparison, the accuracy agreement scores for Google Gemini were 0.205 (slight agreement) for obstetrics and − 0.08 (no agreement) for gynecology.

## 4 DISCUSSION

This study examined ChatGPT and Google Gemini's ability to give accurate responses to patients' questions about common obstetrical and gynecologic topics. For the 10 topics chosen, all responses from both models met the acceptance criteria of at least “adequately complete” and at least “more correct than incorrect”. A Wilcoxon signed‐rank test was used to compare scores between the two models, and confirmed a significant performance gap favoring ChatGPT for both completeness and accuracy in obstetrical topics. In contrast, in gynecologic topics, ChatGPT significantly surpassed Google Gemini in completeness, but Google Gemini significantly surpassed ChatGPT in accuracy.

Our findings align with single‐model studies in obstetrics, where ChatGPT answered perinatal questions accurately, but occasionally missed key clinical nuances.^13^ Comparable accuracy has been reported for LLM guidance on prenatal questions such as hypothyroidism, though readability often remains at a college level.^14^ Additionally, Barbosa‐Silva et al.^15^ found that ChatGPT did not provide answers in line with current guidelines when asked about urinary incontinence, suggesting an issue with both accuracy and completeness. However, only 2% of the initial literature on ChatGPT focuses on obstetrics and gynecology, indicating a significant gap in the research.^16^ Patel et al.^17^ highlight the improvements that AI brings to diagnostic precision, prediction of pregnancy‐related issues, and tailoring of treatment strategies. Elbiss and Abu‐Zidan^18^ show that AI prediction tools were more accurate than gynecologists in cases surrounding endometriosis and acute abdominal pain. Another study completed by Muluk^19^ found that ChatGPT and Google Gemini excelled in omitting false information and maintaining relevance, respectively, when answering birth control questions. Although neither model was flawless in our study, ChatGPT generally delivered more comprehensive responses, and Google Gemini provided more accurate answers. Physicians need to understand the strengths and limitations of these models, as a patient's or physician's preferred model can influence their understanding and treatment.

In addition, although both ChatGPT and Google Gemini provided citations for most answers, the accuracy and legitimacy of these references remained unclear. The absence of traceable evidence makes it difficult for patients and clinicians to confirm guideline concordance. These chatbots tend to fabricate or falsify information and lack citations.^20^ This allows easier fabrication of data. The Office of Research Integrity of the US Department of Health and Human Services defines fabrication as the creation of data, whereas falsification is the manipulation of research materials resulting in inaccurate representation.^21^ The literature refers to this phenomenon in AL models as “hallucinations”.^22^ Athaluri et al.^22^ found that among 178 references cited by ChatGPT, 69 lacked a DOI, and 28 of those were determined to be entirely fabricated. Another study conducted by Else^23^ asked ChatGPT to generate 50 artificial abstracts for review by medical researchers, who identified 32% of these as real.

**FIGURE 5:**
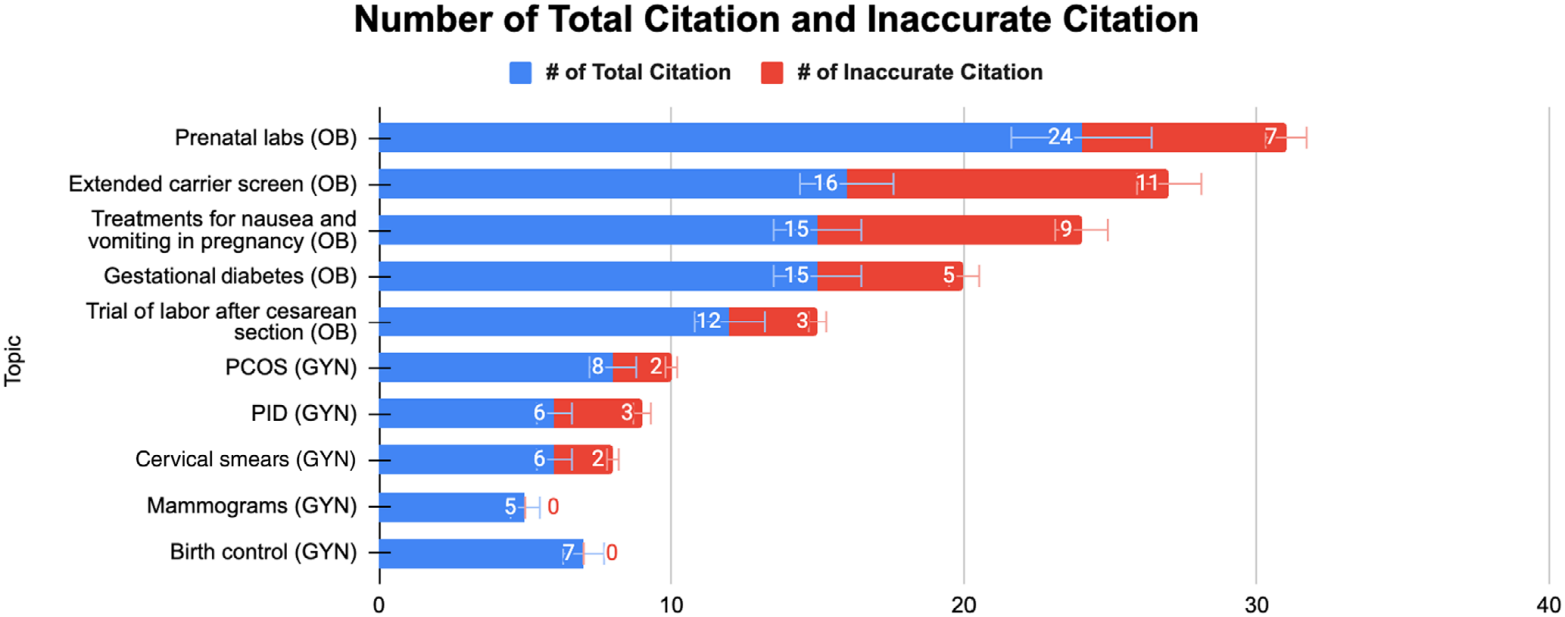
ChatGPT's inaccurate citations versus total citations.

In our study, ChatGPT and Google Gemini's information sources were unclear, potentially disconnecting patients from sources and hindering research. The two models were asked to list the resources used to generate the information. ChatGPT provided a total of 114 references, of which 42 were either fabricated or inaccurate, resulting in a 36.8% inaccuracy rate. Conversely, when Google Gemini was prompted to share the resources it used, it responded, “I apologize that I cannot directly fulfill your request for a traditional reference page from my own ‘sources’, but I hope the explanation and the suggestions for reputable organizations are helpful for your research.” and supplied links to home pages such as The Mayo Clinic and the American College of Obstetricians and Gynecologists. These results highlight the risks of LLMs addressing medical questions without verification from a healthcare provider. This deception endangers healthcare providers and researchers, but also ultimately harms patients. False data and misinformation can lead to misguided decisions and complicate the integration of AI in health care. A visual representation of the false citations provided by ChatGPT, categorized by topic, is displayed in Figure 5.

Both models generated lengthy answers to most questions without giving sufficient thought to the inherent complexity of the topics. The sheer volume of information in such an extensively detailed response creates confusion for patients seeking clarity. For instance, a straightforward inquiry regarding the management of gestational diabetes prompted detailed explanations of nutritional counseling, glucose‐monitoring protocols, oral hypoglycemic agents, and insulin treatment plans. Additionally, when faced with the question concerning the selection of appropriate birth control options, the answers provided by both models were notably lengthy and detailed. Although the information was complete and accurate, it lacked contextual nuances, which may lead to greater confusion compared with the baseline. In the absence of context, a patient could misinterpret these options as equivalent self‐directed alternatives, which could potentially postpone formal care.

Furthermore, AI's lack of emotional intelligence during medical discussions has become a concern. Vzorin et al.^24^ point that while LLMs are capable of identifying emotions, they lack the motivational aspect of emotions. Previous work demonstrates that emotional framing improves patient understanding and adherence.^25^ Yet, LLM responses in our study read as neutral and information‐dense, with limited expressions of reassurance or shared decision making cues. Some researchers argue that synthetic empathy may falsely amplify patient trust, but others warn that its absence could widen comprehension gaps for non‐native English speakers or culturally diverse populations.^26^

This study sheds light on emerging LLM technologies. However, it has some limitations. The Likert‐style questions are subjectively graded, and the ratings can vary from one reviewer to another. This limitation is evident in the suboptimal interrater reliability, indicating inherent subjectivity. Expanding the number of reviewers and increasing diversity may provide more comprehensive data and improve generalizability. Furthermore, AI models have biases in their training, so may exhibit limitations with rare conditions or diverse populations. Future studies should also explore these gaps to contribute to the standardization of AI in medical care and continue to explore its strengths and weaknesses as they evolve.

In conclusion, the present study indicates that both ChatGPT and Google Gemini can generate responses that are typically accurate and comprehensive on common OB/GYN topics. Nevertheless, even minor variations in performance can have significant implications when patients rely on these resources for medical comprehension. Although LLMs can act as adjuncts to medical advice, they are not currently capable of substituting for it. The development of these models in OB/GYN care should be guided by careful assessment, clinician participation, and ongoing research aimed at promoting equity, safety, and transparency. For the time being, physicians need to continue emphasizing the importance of verifying information, whether from LLMs or other online sources.

## AUTHOR CONTRIBUTIONS

MW contributed to the formal analysis, methodology development, writing—original draft preparation, and writing—review and editing. AA contributed to the formal analysis, methodology development, writing—original draft preparation, and writing—review and editing. RS and FA contributed to writing—original draft preparation. RC and LQ contributed to rating answers, writing—original draft preparation, and writing—review and editing. NS contributed to the conceptualization of the study, formal analysis, methodology development, supervision, and writing—review and editing. All authors have read and approved the final version of the manuscript and agree to be accountable for all aspects of the work.

## FUNDING INFORMATION

This study received no external funding.

## CONFLICT OF INTEREST STATEMENT

The authors have no conflicts of interest.

## Supporting information


**Data S1.** Authorship‐change‐form_IJGO.

**Data S2.** Supplemental Table.

## Data Availability

Data available in article supporting information.
